# Exploring the Hidden Curriculum’s Impact on Medical Students: Professionalism, Identity Formation and the Need for Transparency

**DOI:** 10.1007/s40670-020-01021-z

**Published:** 2020-07-24

**Authors:** Megan E.L. Brown, Oluwafemi Coker, Annabel Heybourne, Gabrielle M Finn

**Affiliations:** Health Professions Education Unit, Hull York Medical School, University of York, John Hughlings Jackson Building, University Road, Heslington, York, YO10 5DD UK

**Keywords:** Hidden curriculum, Professionalism, Professional identity, Medical students, Anticipatory socialization

## Abstract

The hidden curriculum within medical education has been a topic of recent debate. Consensus opinion regarding the continued relevance of this term, what constitutes the hidden curriculum, and the nature of its impact do not exist. Further research is required to contribute to this debate. This work sets out to investigate which factors beyond taught cognitive knowledge influence medical students in clinical and educational environments and examine how this occurs. Semi-structured focus group interviews were conducted with 39 students from one UK medical school. Fourteen faculty were interviewed individually to triangulate data. Data were analysed using constructivist thematic analysis, informed by grounded theory convention. The presence of the hidden curriculum was clearly demonstrated, acting through role modelling, organizational culture, stereotyping and professional dress. Mentioned frequently were the influences of the hidden curriculum on student professionalism and identity development. Professionalism was perceived as being negatively impacted by the hidden curriculum and seen as an imposition from senior faculty to control students. Students believe medical identity formation begins prior to medical school, in a process known as “anticipatory socialization”, a previously unstudied identity transition. Students felt covert institutional agendas negatively impacted their identity, pushing them further from the identity their institution was encouraging them to acquire. Key messages for educators include the need to explore the hidden curriculum through discussion with students. Improving transparency of organizational culture may allow students to interpret institutional agendas in the way institutions formally intend, reducing orthogonal interpretations of organizational culture and subsequent impact upon identity formation.

## Introduction

Medical school is designed to equip students with the knowledge and skills required to become a doctor. Whilst some sources, such as medical school and national guidance [1], do aim to increase student awareness regarding what is expected of them upon graduation, guidance on the reality of becoming a doctor is often lacking. The practicalities of life as a clinician are typically observed and learnt through experience in the clinical world of medicine—this informal, and often unintended, learning is often termed the hidden curriculum [2].

The hidden curriculum “deals with the tacit ways in which knowledge and behaviour are constructed”, operating outwith of a medical school’s specified formal curriculum [2]. It consists of a set of influences acting upon students as a result of their presence within a specific organization [3]. These multifaceted influences operate within a student’s social environment and include “corridor conversations”, role modelling behaviour and assessment regimes [4].There is contemporary debate surrounding the definition of the hidden curriculum, with some viewing the term as the unwanted aspects of becoming a doctor [5] and others asserting the hidden curriculum includes positive interactions [6] and can offer benefits to both students and faculty. For the purpose of this research, the definition of the hidden curriculum will be taken as it has been described at the start of this paragraph. This definition is without positive or negative connotations, in order to ensure findings are grounded in participant experience.

Debate also exists as to the continued relevance of the concept of a “hidden curriculum”. Although the concept has become commonplace in educational discourse, views exist such as those voiced by Macleod, that the term has become problematic [7]. The term “hidden curriculum” is broad in definition, including varying “depths of hiddenness” [7], which may prevent clear academic discourse [8]. Hafferty and Martimianakis argue against this view, putting forth the breadth of the term allows for conceptual richness and theoretical diversity in investigation of this complex concept, which cannot be reduced to a singular understanding [9]. Despite this, they acknowledge the need for clarity regarding which elements of the hidden curriculum are to be examined. This will be actively considered in the research questions of this work.

The influences operating within the hidden curriculum are held to impact students in several different ways. Focus has largely been given to the negative impact of the hidden curriculum upon medical students. These impacts include the socialization of medical students to manifest less professional or moral behaviours [10–12] and damaging effects upon the professional identity formation of medical students leading to emotional dissonance [13]. Much less research has been done concerning the potential positive impacts of the hidden curriculum upon medical undergraduate students. Some work casts doubt upon findings that the hidden curriculum serves as a purely negative influence on student professionalism and identity formation [14] and demonstrates benefits. These benefits include student internalization of robust medical ethics as a professional value [15]—indeed, recent research reveals some faculty attempt to deliberately manipulate the hidden curriculum to teach concepts such as professionalism “by stealth” [16]. Further to this, some hold a positive hidden curriculum surrounding professional identity formation may be fostered if the complexities of clinical practice are made explicit [17]. Given ongoing debate, additional work aiming to highlight the factors, and nature of these factors, beyond taught cognitive knowledge that impact medical students is appropriate to contribute to generating an academic consensus.

Given this, and the fact that most research evaluating the impact of the hidden curriculum has been done from the view of medical educators [18, 19], further investigation of the hidden curriculum’s impact upon and with medical undergraduate students is essential. This work explores factors contributing to or forming the hidden curriculum within one UK medical school from the perspective of medical students. It questions the nature of the hidden curriculum’s impact and details the mechanism by which this impact occurs. Ascertaining this impact could prove difficult—indeed, by its very name, this type of curriculum is “hidden” and therefore not easily evaluated [20]. In order to determine the impact of the hidden curriculum upon medical students, focus group data were also triangulated with individual faculty interviews, to deepen data analysis.

## Materials and Methods

### Epistemological Considerations

The paradigm of this work is constructivism, a world-view recognizing that reality is multiple in nature and socially constructed by individuals [21]. Constructivism is a way of approaching research that acknowledges the subjectivity of reality and knowledge [21]. This understanding influences the research approach selected and informs interpretation of participant data. Given that students experience the hidden curriculum subjectively as a social process [3], constructivism is an appropriate paradigm to direct and guide this work. The qualitative methodology selected for use within our paradigm is constructivist thematic analysis, informed by grounded theory convention and acknowledging the active role of the researcher (and subsequent subjectivity) in data generation and analysis [22–25].

### Research Questions

This work has three central research questions, outlined below. The first question is intentionally broad, in order to embrace the acknowledged breadth of the concept of the hidden curriculum. The research team hoped this question would facilitate investigation of both the hidden curriculum within student clinical experiences and regarding educational experiences. In this way, we define the scope of our research regarding the hidden curriculum by stipulating which contexts within medical education will be examined. This aligns with Macleod’s adaptations of Vallance’s three dimensions of critical thinking regarding the hidden curriculum. We expand upon this specificity in our secondary research question, investigating student definition of the term “hidden curriculum” and their application of this term to their own experience. The third research of this work centres around perceptions regarding whether the hidden curriculum can be taught and assessed, given contemporary debate regarding whether the hidden curriculum can be made visible.

1. What factors beyond the formal curriculum (e.g. including taught cognitive knowledge) impact on medical students within clinical and educational settings?
2. What does the term “hidden curriculum” mean to medical students? How do they apply this definition to their own experiences to identify factors that have influenced their practice, attitudes, knowledge and behaviour (i.e. learning)?
3. How do students and tutors conceptualize the relationship between teaching, assessment and the hidden curriculum?

### Study Design and Data Collection

Ethical approval was granted by the Hull York Medical School (HYMS) Ethics Committee. Semi-structured focus group interviews with 39 medical students were undertaken by two authors (OC and AH) within HYMS. HYMS runs a five-year undergraduate medical programme, with an option for interested students to undertake a year of intercalated study following second or third year. Each focus group contained 4 or 5 students. In addition, semi-structured individual interviews were conducted with 14 active medical school faculty, as a way of triangulating student data. All interviews were audio recorded and transcribed verbatim by the research team.

Students were recruited from all years of the undergraduate MBBS course and, through the utilization of theoretical sampling, equitable distribution of students from all years of the course was achieved. Theoretical sampling was used in this research with the hope of gaining a more nuanced understanding of the hidden curriculum through participant experience and representative of the wider student and tutor body [26].

Participation in this research was voluntary. Recruitment of students occurred electronically via email and posts within Blackboard, the Digital Learning Environment used by medical students. Recruitment of staff occurred electronically via email and through the use of snowball recruitment. In order to generate meaningful results, inclusion criteria were set for staff requiring them to be involved in the teaching of students at least once a month. Interested students and staff responded to the advertised research via email and were subsequently sent the study participant information sheet. After an appropriate amount of time had lapsed to read the information sheet, interested parties were emailed the study consent form. Prior to study commencement, all researchers engaged in individual reflection and subsequent group discussion concerning reflexivity to consider their own perspectives in more detail. Two authors are medical education researchers (GMF and MELB); one is a medical student (AH), and one author, although now a junior doctor, was a medical student at the time of the research (OC).

Both the semi-structured focus groups and individual interviews were conducted iteratively. Group semi-structured interviews allowed us to understand a range of individual views concerning the hidden curriculum and explore contrasting student opinion [27, 28]. A focus group topic guide was produced by the researchers, derived from previously published studies regarding the hidden curriculum and allowing for a semi-structured approach. The focus group topic guide detailing the spines used to facilitate discussion can be seen in Table 2, in the appendix to this work. Medical school tutors were offered individual semi-structured interviews, instead of group interviews in order to increase participation [19, 29]. At HYMS, tutors are also practicing clinicians and so often have fixed clinical commitments that scheduling group interviews around is difficult. Given this, tutors were offered individual interviews to allow them to practically participate. For tutor interviews, interviewers were provided with an interview guide, containing open question stems to stimulate discussion and encourage a conversational interview style [28]. The interview guide for tutors is presented in Table 3, Appendix section.

### Data Analysis

Open, focused and theoretical coding of interview and focus group transcripts were carried out by all members of the research team in order to deepen analysis. Coding was done in iterative cycles with constant comparison. Following initial open coding, the research team met to discuss content groups and resolve any disagreements regarding groupings. Memo-writing was utilized throughout by each researcher. Comparison of memos assisted in content grouping. Focused coding and theoretical coding were used to generate a rich thematic picture detailing factors within the hidden curriculum impacting upon students, alongside the nature and mechanism of these impacts. Concurrent with open coding, the research team met regularly to discuss the informational power of this research with final student and tutor participant figures being decided upon as sufficient to produce transferable results [21].

## Results

### Themes

One hundred ninety-nine descriptive open codes were initially identified from all interview transcripts. These were collapsed into 65 final open codes. Student and tutor transcripts were coded iteratively and together to provide a more in-depth analysis. Ten sub-themes and three major themes were identified. These are detailed in Table 1, below.

**Table 1:** Open codes, sub-themes and major themes. Major themes are listed at the top of the table, with the sub-themes of each major theme listed vertically underneath. Underneath each sub-theme is a list of open codes that generated the sub-theme

Major themes:	Professionalism	Professional identity formation	Attempting to illuminate the hidden curriculum
Sub-theme 1:	What is professionalism?	Personal identity	Teaching
Open codes:	Defining professionalism	Innate identity	Can you teach the hidden curriculum?
	Context	Professionalism means harm	Peers
	Importance	Personal identity is human	Immersive experiences
	Role of society	Personal vs professional identity	Interpreting teaching
	Professionalism is a choice		Traditional teaching methods
	Grey areas		Oral culture
	Being unprofessional		Online resources
Sub-theme 2:	Acquiring professional attributes	What is professional identity?	Assessment
Open codes:	What are professional attributes?	Medical identity is special	Should we?
	Teaching professionalism	Timeline of identity development	Exams impact learning
	Clinical experience	Defined by the hidden curriculum	SJTs
	Impact of the hidden curriculum	Defined by PBL	Preparation for practice
	Detachment	Medical identity is taxing	Senior exams
	Behaviour modification	Identity and respect	
		Clothing	
		Professionalism and identity	
Sub-theme 3:		Help and the hidden curriculum	Role modelling
Open codes:		Communities of practice	Positive role modelling
		Responsibility	Negative role modelling
		Peer network	Impact
		Reflection	Uses
		Clinical exposure	
		Junior doctors	
		Coping	
		Identity role modelling	
Sub-theme 4:		Harm and the hidden curriculum	Awareness and knowledge of impact
Open codes:		“Groupishness”	Practice implications
		Service pressures	Seniority improves awareness
		Stereotypes	“Exploiting” the hidden curriculum
		Dissonance	Equal impact to formal curriculum
		Selection processes	Need
		Enforced identity	Advantages
		Organisational management	Disadvantages
		Identity role modelling	

#### Major Theme 1: Professionalism

### What Is Professionalism?

Both students and tutors spoke about defining professionalism within the hidden curriculum. For students, professionalism was viewed in a largely negative light.

Professionalism… is the things we shouldn’t do.

(Student 5)

Tutors acknowledged students acquired professionalism gradually within the hidden curriculum, as their awareness of the hidden curriculum increased.

I would loosely see it as a set of behaviours, attitudes that students develop as they move along that trajectory from being a student to being a junior doctor.

(Tutor 4)

### Acquiring Professional Attributes

Discussing professionalism and the hidden curriculum involved participants detailing the acquisition of professional attributes. There was debate regarding what professional attributes are. Tutors recognized respect for patients as key.

It’s an ability to respect your patients I think that’s absolutely key…

(Tutor 5)

Although students acknowledged the importance of respecting patients, they tended to focus more upon the modification of behaviour to portray professional attributes, in opposition to the importance of attributes in and of themselves. Professional dress was identified as important and students lamented the loss of white coats. Students believed white coats portrayed professionalism and strengthened medical professional identity.

Dress code is an interesting thing…you see people coming with shirts stained, jeans, and you think and if I went to my doctor that’s not what I would want them to look like…I think that’s definitely changed a lot especially with the drop of white coats…its identifying doctors in the hospital as well I think is difficult. Could it be a pharmacist or a psychologist or whatever whereas there used to be a very defined identity of you are the doctor, you are the patient...

(Student 23)

There was debate as to whether professionalism could be taught within the formal curriculum, with it thought to be most useful in early formal instruction.

I think early on you need to alert them to pitfalls.

(Tutor 1)

I think a good framework can definitely be taught…you can definitely teach good fundamental principles of professionalism and then from that, you can build on it yourself.

(Student 13)

It was recognized professionalism acquisition occurs largely within the hidden curriculum with students learning from experience and tutor behaviour.

You can’t really teach us everything, so you learn by picking it up from others like junior doctors.

(Student 25)

#### Major Theme 2: Professional Identity Formation

Both students and tutors spoke about professional identity formation occurring underneath the umbrella of the hidden curriculum.

### Personal Identity

Students and tutors described how personal identity is impacted by the acquisition of medical professional identity.

Doctors feel they are a doctor even when they are not at work.

(Tutor 2)

I think for me one of the most important aspects is setting aside your own values and ideas and taking up the profession’s instead.

(Student 13)

Students experienced conflict between their personal identities and emerging professional identity. This often led to a shift within student personal identity and behavioural changes facilitating acceptance within the medical field. This conflict was not witnessed within tutors, suggesting seniority and time spent within the hidden curriculum may resolve conflict.

I really struggle…I definitely try to be more professional in everything I do rather than just in hospital. I think that can sometimes impact what I do in my life and it can sometimes feel a bit restrictive because I do moderate myself a lot.

(Student 1)

You see it in certain people; say they want to join the surgical society, some people adopt a certain persona…you get a sense of where people put themselves forward in a certain way, they get more accepted.

(Student 36)

### What Is Professional Identity?

Defining professional identity was described when discussing the impact of the hidden curriculum. Medical identity was perceived as “special”, affording doctors respect.

You definitely get treated differently by members of the public…they’ll tell you their life story just because you’re a medical student. You definitely do have that identity sort of thing in the profession.

(Student 6)

It really discredits [supervisors] if they like don’t really have a professional identity, you wouldn’t necessarily go to them with a question…you’d want to go to who someone who knew what they were doing.

(Student 11)

Professional identity was identified as being influenced by the hidden curriculum.

The hidden curriculum… its everything that we don’t formally think about that influences the development of identity.

(Tutor 6)

There was debate over the timeline for the formation of medical student professional identity.

Tutors believed professional identity formation could only occur when practicing as a doctor, fully immersed within the hidden curriculum of the workplace.

Real identity is developed after you finish medical school.

(Tutor 5)

However, most students believed their medical professional identity began to form prior to medical school admission, when they decided to become medical applicants. Early identity changes were most likely to take the form of altering behaviour to adopt medical professional attributes, such as moderating social media usage.

Interviewer: So would you guys consider censoring what you were doing say on social media to maintain your professional outlook?

Student 10: Yes. I already have. I think it starts as soon as you become a medical student, if you’re, well if you’re like-minded.

Student 9: Prior to becoming a medical student…

Student 11: Yeah, it’s being careful about what you post isn’t it? And… what other people post of you.

Student 9: Yeah, that’s true, you can’t always censor that I guess, so it’s censoring yourself in reality…

Student 10: So it’s not just censoring what you post, it’s censoring actions as well.

(Students 9, 10 and 11)

I realised I wanted to do medicine…after I realised, my mindset changed, I thought I will never do something that would be regarded as unprofessional…

(Student 28)

Students also recognized the presence of professional identity transitions within the hidden curriculum. The identity transition most frequently commented upon was that that occurs upon the commencement of clinical exposure.

That was an abrupt transition from having been a student for what 6/7 months with no clinical activities whatsoever to writing in the notes on the ward round the next day… immediately I felt almost like a different person. It felt like a very abrupt transition. I felt like a different person walking around the hospital.

(Student 15)

### Help and the Hidden Curriculum

It is clear from the above that clinical exposure aids medical identity acquisition. Dissecting this, involvement within a medical community of practice strengthens identity formation and assists learning. Junior doctors were valued the most as having recent hidden curriculum knowledge.

When you’re on a ward and someone gets you to be part of the team, you learn more.

(Student 24)

Junior doctors will teach what we need to know; focusing on the important knowledge required for finals.

(Student 37)

Students recognized not just positive experiences within the hidden curriculum could assist in identity acquisition but, given appropriate interpretation of the hidden curriculum, negative experiences could also benefit.

So, you’re in a clinic with someone and let’s say they’ve spoken to a patient and you feel that maybe that was a bit abrupt…. X University is quite good at teaching us how to do reflection, so by seeing that in person you’ll inherently go away and think how you would have acted differently and…you alter the way you behave.

(Student 12)

However, interpretation of any negative hidden curricula experiences can differ between individuals and instruction in reflective practice is key to facilitate appropriate interpretation.

I think there are lots of different ways to interpret it…different people will get different things out of each activity.

(Student 19)

### Harm and the Hidden Curriculum

Although negative experiences within the hidden curriculum can be useful, they also have the potential to harm student identity formation.

Medical students acknowledged medical admission processes could unify student identity and reduce personality variety.

Student 21: One of my GPs made the comment, just like oh you guys are a lot less, erm, you’re all normal nowadays, like back in my day there used to be a lot more characters in Med School… Student 20: What does he mean by characters? Like … Student 19: I think it’s just more eccentric people. (Students 19, 20 and 21)

No views were expressed as to whether students thought selection unification would have positive or negative implications upon identity formation, although students did recognize that institutions pushing specific professional identities upon students later in their training, often as a result of wider institutional agendas, pushed them further from the acquisition of that identity.

Student 3: I think that’ll become a bigger issue as well in the next few years with the opening of new medical schools that are specifically designed to retain GPs in those areas and those medical schools lack the anatomy facility and things like that because they are specifically designed for that. I think that’s very difficult for people that go there and have an experience if they want to develop a professional identity of being a specialist in whatever field.Student 2: I don’t mind being an X University student but we get told a lot…it’s a good GP college and it’s just made me want to be the exact opposite and I feel like that has that effect on a lot of us. It’s like you get told so much that ‘oh you’re just going to be a GP’ it’s like no, I’m going to do something better. (Students 2 and 3)

Negative role modelling and institutions failing to manage this was identified by tutors as an element of the hidden curriculum particularly harmful to identity formation.

…there are some really difficult people who behave badly but who are managed within organisations in a way that doesn’t address it. I would see that as one of the biggest problems within the hidden curriculum…I think we can all think of [very senior] people… who behave… appallingly but no one in the organisation can touch [them]... You might get the message as a student…[that] that’s the way to behave if you want to get to the top.

(Tutor 4)

Students generally thought themselves able to counteract negative role modelling, although some acknowledged not everyone may be able to mitigate negative effects upon behaviour and identity.

Student 13: I mean you also just copy people, what your seniors do to some degree, I mean it’s, it’s mimicry of, of your GP, of your consultant, of your peers … it’s just learning from example. Student 14: Yeah, there is plenty when the example’s bad. Student 12: Yeah, but usually you can make a reasonable judgement whether that example is bad or not. Student 14: Not if you’ve got bad judgement.

(Students 12, 13 and 14)

Stereotyping by others was identified by students as prevalent and harmful.

I don’t think there is a medical job where you’re not going to get some flak from somebody: if you’re a nephrologist you’re a nerd if you’re a psychiatrist you’re not a real doctor.

(Student 3)

For me as a girl, by hearing people going like, you know, about orthopods, like actually now I’m thinking about it, maybe I could be an orthopaedic surgeon... But in my head, it was never going to be an option because girls aren’t orthopaedic surgeons.

(Student 2)

The harmful effects of the hidden curriculum caused negative identity dissonance within students. Students spoke about identity dissonance without awareness of the phenomenon, often illustrating a clear divide between their personal and professional selves through the use of clothing to symbolize switching between poorly integrated identities. Here medical professional identity is something to be worn, that they do not yet possess in their own right.

I feel like when I go on placement, I have the placement clothes that turn me into a medical student, so I act differently.

(Student 5)

#### Major Theme 3: Attempting to Illuminate the Hidden Curriculum

Students and tutors identified several ways in which they attempted to illuminate or uncover the hidden curriculum.

### Awareness and Knowledge of Impact

Increasing both student and tutor awareness of the hidden curriculum was identified as crucial in tackling the issues it could pose.

It’s unavoidable, it’s something that students and tutors need to be aware of, that it impacts on learning.

(Tutor 10)

Pre-clinical students had a particularly poor understanding of what the hidden curriculum was.

I don’t really know what you mean by hidden curriculum.

(Student 18)

Clinical students displayed more meaningful views of the hidden curriculum.

I suppose what medical school teaches you is how to pass and then everything else is how to be a doctor.

(Student 10)

One explanation for this is that the hidden curriculum has a larger impact upon, and relevance for, senior students. This impact is heightened by the prospect of practicing as a junior doctor.

It’s helpful because you’re learning things you will need in the real world…

(Student 33)

Senior students identified the hidden curriculum as having as least as large an impact as the formal curriculum. Awareness of its impact and benefits made students more likely to try and uncover the hidden curriculum themselves.

I think it is just as important because you can know the most information but be the worst doctor by not learning the stuff that isn’t examined.

(Student 39)

You could be helped by other people, but you’ve taken the initiative to find out and then it has an equal impact on your learning as other formally taught things would do.

(Student 27)

However, students also expressed concern at uncovering clinical aspects of the hidden curriculum, due to the need for the previously mentioned individual interpretation of its impact.

The bad thing is, it depends completely on who you’ve got around you…that relies on your personal judgement a lot...

(Student 25)

### Teaching

We have already unearthed debate surrounding professionalism teaching. Debate is also seen more generally as to whether the hidden curriculum can be taught. Most individuals thought the hidden curriculum could not be taught and believed knowledge and skills forged within this were acquired through experience.

I think looking at all these things that are hidden, there’s loads of things that you can’t explicitly teach, I think you have to actually sort of see it or do it yourself to actually learn …

(Student 7)

Some of it you only pick up by being immersed in that environment.

(Tutor 1)

Students expressed a preference for baseline knowledge to be built on didactic teaching and then solidified by experience within the hidden curriculum. Tutors also expressed preferences for an integrated approach, combining formal, informal and hidden curricula.

I think…if you get teaching but also experience at the same time, so you can learn about confidentiality and you can actually see it in practice and that kind of like marries the two together and it helps you actually be able to do it yourself.

(Student 6)

In terms of actually teaching it, it needs to be woven through everything that they are doing…

(Tutor 4)

Students referenced a well-developed oral culture surrounding educational hidden curricula that acted as a form of teaching or instruction.

A lot of us were guided by the older years and it worked. They’d say ‘go and use this book…this is important, but this isn’t very important’.

(Student 27)

However, there was concern regarding this oral culture—some students felt unable to trust unstandardized information, and some saw it as representing a deficiency within the formal curricula of the medical school.

I take [hearsay] with a pinch of salt. In first year, I paid quite a lot of attention to it…these days I listen to them and think ‘yeah, maybe but actually I’m just going to learn what I think is important’.

(Student 34)

Obviously, that’s always going to be word of mouth as students, but I do think the school should also be overt about it…the medical school shouldn’t rely on that as part of the hidden curriculum.

(Student 2)

### Role Modelling

Role modelling was frequently detailed as a key element of the hidden curriculum. There is significant interplay between role modelling and “teaching” of the hidden curriculum, given that so much of instruction within the hidden curriculum takes place within a clinical environment. In this way, students recognize role modelling as having a positive impact on their learning experience.

We had a renal consultant who was really happy to teach… that makes you want to learn that subject.

(Student 24)

Given the impact of role modelling, negative role modelling can prove harmful. We have already demonstrated negative role modelling within the hidden curriculum can prove harmful to student identity acquisition. Building upon the development of this identity, negative role modelling experiences can influence student speciality choice.

Student 4: There’s definitely a lot like placements as a medical student, you get put with a bad consultant for obs and gynae so you don’t like obs and gynae sort of thing…Student 1: Well like you were saying earlier about one bad consultation can ruin a doctor’s career, one bad experience of a specialty can ruin a specialty.

(Students 1 and 4)

I think people take a lot of direction from their tutors when making decisions on what they may want to specialise in.

(Tutor 4)

As well as role modelling impacting upon experience within the hidden curriculum, role models can act directly to help students illuminate the hidden curriculum. Some tutors suggested this is achieved through explicitly addressing the hidden curriculum and encouraging students to reflect upon its impact.

That hidden curriculum needs to remain there in the way it is, the soft background socialization culture thing, but also needs to become explicit of the tasks we are asking of our students.

(Tutor 13)

My view is that if you discuss openly with students, students can start to see and look on their own behaviours and attitudes. You hope that whilst you can’t teach it, you hope they can reflect on themselves and adjust accordingly. It requires students and doctors to be open and reflective and that’s the bit that you can encourage.

(Tutor 12)

### Assessment

There is debate as to whether we should formally assess the hidden curriculum within medicine and, if we should, how this could happen.

Students voiced concerns over attempting to assess the hidden curriculum as there is little parity between experiences within the hidden curriculum.

…the problem is on placement, every hospital and every department is different so it’s quite hard.

(Student 24)

Both students and tutors recognized that examinations influence student behaviour which may impact upon learning.

You do neglect things that you’re told will not be in the exams...

(Student 34)

Students and tutors both identified the hidden curriculum surrounding institutional examinations which, if not explicitly addressed prior to assessment, causes difficulty and distress.

There’s a lot of discrepancy as to what we need to know for the clinical exams…I’m redoing 5th year…a lot of issues we were having was about what they wanted.

(Student 25)

The first two years… they are told this message that everything that’s in the curriculum is up for assessment, but I don’t sometimes think that they always hear that. Maybe we should be reinforcing that message a bit more.

(Tutor 7)

Students felt assessments were not well aligned with actual clinical practice. This lack of alignment contributed to uncertainty surrounding clinical examinations and acted as a source of the hidden curriculum.

If you do the thing the way you’ve been taught on the ward, you will fail the exam. You’ve got to learn both because you need to pass your exams and become a practical doctor.

(Student 34)

In medical school you learn how to pass the exams, whereas on the wards you learn how to actually be a doctor… it’s been hard to have to learn both.

(Student 31)

It’s like, on one hand, FY [Foundation Year] doctors say you need to know how to do discharge letters… but I’ve never had time to really learn because that’s never going to come up in exams.

(Student 17)

## Discussion

### Key Messages

Medical student and tutor views reveal much about the impact of the hidden curriculum. The findings of the work revolve largely around student professionalism and identity formation. Yet, how do these findings fit with what is already known?

Students and tutors acknowledge that the development of professionalism within the hidden curriculum can detract from medical student identity. Professionalism was viewed negatively; many believe adopting medical professionalism involves erasing one’s own identity, which coincides with Erikson’s belief that the suppression of one’s personal identity is necessary to affect identity change [30]. Given this, discomfort in adopting professional attributes here could represent a normal part of incorporating medical professional attributes into one’s professional identity. However, a greater depth of evidence has emerged demonstrating identity suppression can cause identity dissonance and significant emotional distress [5]. Concerns also exist that a hidden curriculum encouraging the adoption of professional attributes which are defined in a “top-down” fashion restricts students [31] and risks propagating discrimination. The concept of medical professionalism is seen as upholding the views of the most powerful within the field—typically white men—and diverse students are discouraged from freely expressing identities that authority figures view as “non-traditional” [32]. Our work adds weight to these emerging claims—medical students find the hidden curriculum defines professionalism in a heavily boundaried, “top-down” fashion and this negatively impacts upon their sense of self and subsequent behaviours, where they feel they need to become overly restrictive. The definition of professionalism within the hidden curriculum needs addressing—without explicit evidence of institutional work to address student concerns, professionalism risks being viewed by students as “something used more to control… behaviour than to stand as a core occupational value” [33].

During medical school, students progress through a series of identity transitions [34]; however, what is less well discussed, and a principal finding of this work, is the notion that medical professional identity formation begins prior to admission to medical school. Students overwhelmingly believed their medical identity and conscious alterations to their behaviour occurred as soon as they decided to become medical school applicants. It could, therefore, be hypothesized that the hidden curriculum may be acting even prior to entering medical school. Within wider occupational literature, a concept known as “anticipatory socialization” exists, where preconceived notions of a profession can have profound effects on later values and behaviour [35]. Cavanagh et al. demonstrated medical students understand the expectations of their profession more so than students on other university courses, with this understanding likely developing prior to course commencement [35]. Therefore, medical students are likely to be affected by anticipatory socialization more than other developing professionals. Despite this, very little research concerning anticipatory socialization’s impact upon medical identity formation exists [36, 37]. This work adds weight to previously tentative claims that medical students undergo a period of anticipatory socialization prior to university entry, in which medical professional identity begins to form. The hidden curriculum of medicine begins to act within this period, shaping students before they even set foot within a medical school. What this hidden curriculum entails is likely different for each student, as they hold diverse experiences and views of the medical profession before they enter into a medical institution with a more standardized curriculum and, therefore, arguably more predictable hidden curriculum. Although entry to medical school undoubtedly represents a professional identity transition, students do not approach this transition as a blank professional canvas—indeed, deciding to apply to medical school may represent a previously unidentified, additional identity transition.

It is well held that positive student experiences within the clinical environment act as a hidden curriculum in assisting with professional identity formation [38–40]. What has not been well established, however, is that benefits can be offered through negative student experiences within the hidden curriculum. The hidden curriculum operates within the real world of medical practice; it is not a simulated environment and is, therefore, impossible to fully control. Given this, students are liable to encounter negative experiences during immersion within the hidden curriculum. Students within our study felt such negative experiences strengthened their desire to do better and improve their own behaviour, given appropriate reflection upon said experiences. An important distinction must be drawn here, however—student mistreatment within the hidden curriculum has been proven to damage student learning [41] and this is not what our findings advocate. Medical education literature has previously demonstrated the damaging effects of negative role modelling [31, 42, 43], and that reflection upon such experiences can negate any lasting damage to student learning, professionalism or identity formation [44]. Previous work has made tentative claims that reflection upon negative experiences in “sense-making” activities could assist students in embodying medical professional identity [43, 45]. Despite this, the focus of the majority of the work done in this area involves avoiding any negative impacts upon identity through reflection [46]. This work goes further, more thoroughly demonstrating that appropriate student reflection upon negative experiences within the hidden curriculum can not only negate negative impacts upon learning but also can impart positives. One could even class negative experiences as a “complexity” of clinical practice that Wald et al. acknowledge require making explicit to create a positive hidden curriculum assisting in identity formation [17]. Undoubtedly, we should train clinicians to increase awareness of the impact of role modelling and support them in developing as purveyors of positive experiences for our students. Yet, even with such training, we have limited power to control the reality of our fraught medical system and cannot, indeed perhaps should not, shelter our students from the reality of medicine. This work offers a new view—a more pragmatic approach needs to be adopted, with institutions improving student awareness of negative role modelling and arming students with the appropriate reflective tools to interpret the negatives of the hidden curriculum in a constructive and beneficial fashion.

Negative effects of the hidden curriculum have been well researched—so much so that some scholars believe the hidden curriculum represents only the unwanted aspects of becoming a doctor [5]. This work echoes previous findings that the hidden curriculum of clinical experiences can act to negatively impact a student’s future speciality choice, as well as damaging learning through negative role modelling and student mistreatment [46, 47]. A more novel finding of this work, however, concerns the damaging hidden curriculum of institutional agendas and organizational culture, often driven by recruitment pressure, upon student identity formation. Students recognized that institutions trying to encourage specific professional identities to assist local recruitment (e.g. through increased exposure to primary care) pushed them further from the acquisition of that identity. Institutional efforts to improve recruitment and retention to traditionally low recruitment specialties seem to be operating in a counterproductive fashion. It is acknowledged one’s identity is influenced by socio-cultural factors and is institutionally situated [48, 49]. Given this, one can logically assume an institution’s agenda will impact upon student identity acquisition. Some view such identity regulation of practitioners by institutions within adjacent fields as a form of organizational control [50, 51]. Concerningly, our students voice experiencing such organizational pressure to conform—institutions must re-evaluate their approach to agenda application, particularly in regard to recruitment and retention initiatives, to ensure their strategy is not acting counterproductively. Lack of institutional transparency regarding higher agendas is seen as hierarchical and as a barrier to professional development within adjacent occupational fields [50]. Improving the transparency of medical school agendas may allow students to interpret any perceived institutional pressures and prevent the alienation of many students.

The participants of this work voiced a desire for the hidden curriculum to be illuminated and assessed to produce better prepared medical graduates, although there was debate over whether this could be achieved. Several scholars have suggested that the vast majority of medical knowledge is tacit, and that the knowledge acquired within the hidden curriculum is no exception [6, 52]. Polyanyi describes tacit knowledge as information one knows but cannot tell [45], with it being impossible for all tacit knowledge to be brought to light [48, 52]. Given this, it would be impossible to fully illuminate and examine all components of the hidden curriculum, although a desire for this was expressed. Educators must make tough decisions regarding the hierarchy of tacit knowledge and decide just what needs to be made explicit in order to aid student identity formation and professional development. The impossibility of highlighting all priority areas of tacit knowledge should also be acknowledged with students, and continual feedback sought regarding the relevance and appropriateness of faculty identified tacit knowledge priorities. This process is currently insufficient and has allowed a divide to develop between final medical school exams and practice as a junior doctor [53]. This work attributes this divide to a failure to prioritize and illuminate tacit knowledge within the hidden curriculum. Further work is needed utilizing both student and educator opinion to develop a priority list of tacit medical knowledge normally imparted by the hidden curriculum that would most benefit from being directly illuminated through teaching and assessment.

### Limitations

One limitation of this study is that it examined the hidden curriculum of a single institution, potentially limiting result transferability. Despite this, we feel our results are transferable to medical students within other institutions nationally and internationally—our sample size is intentionally large and we utilized theoretical sampling to improve informational power. The findings and key messages of this work are relevant to any educator interested in the impact of their institution’s hidden curriculum upon their students, and what they can do to help maximize student benefit.

The composition of the group interviews may also limit this work. Students were assigned to different groups to ensure an adequate mixture and representation of students from different years of the MBBS course, in the hope that this would generate a richer discussion regarding a variety of experiences. However, this method does run the risk of paying inadequate heed to the importance of student identity transitions. Stephens et al. acknowledge three key transitional time periods of medical student identity [34]. The first transition occurs upon entry into medical school, the second upon transition to a clinical clerkship, and the third just prior to graduation. Had the volunteering medical students been grouped so they were in groups with others at the same transitional stage as they were, this may have allowed for a more thorough exploration of these transitions, and the impact of the hidden curriculum at each time point. There is scope for further research investigating the impact of the hidden curriculum upon student identity and professionalism at identity thresholds witnessed within medical students.

## Conclusions

This qualitative study demonstrates that the impact of the hidden curriculum upon medical student professionalism and identity formation is not straightforward. Professionalism was viewed as being mostly negatively impacted by the hidden curriculum, due to erosion of individual identity. Stemming from a lack of clarity and student input in its definition, professionalism was seen as an imposition from senior faculty, used to control students. The impact of the hidden curriculum upon identity formation was more positive, with negative clinical experiences even holding the power to strengthen identity, given appropriate reflection. Students felt covert institutional agendas negatively impacted their emerging identity, pushing them further from the identity their institution was encouraging them to acquire. Students believe medical professional identity formation begins prior to entry to medical school, once they decide to apply, in a process known as “anticipatory socialization”. This likely represents a previously unstudied identity transition and expansion of medical school hidden curricula.

Whilst it is difficult to fully confront or illuminate the hidden curriculum, it is clear current efforts are insufficient, with students perceiving dissonance between their final exams and practice as a junior doctor. Key messages for educators include the need to reveal priority areas of the hidden curriculum through open discussion with students, as well as providing students with the skills to recognize and reflect upon areas of the hidden curriculum they encounter. This work could act as a springboard for discussion with students, as knowledge of where benefit and harm lie within the hidden curriculum is the first step in being able to practically confront it. Educators must make tough decisions regarding which areas of the hidden curriculum are a priority to highlight to students. One key priority area is improving the transparency of institutional agendas, allowing students to interpret institutional pressures and ameliorate negative effects upon the formation of their identity. Overall, the hidden curriculum cannot be avoided. It is a complex concept that is always at work instructing medical students in one way or another. Although it is difficult, perhaps impossible, to uncover every nuance of the concept, we have a duty as medical educators to confront potentially damaging aspects of the hidden curriculum that have become more visible through research, as outlined above.

Areas identified by this study warranting further research include further research concerning the impact of the hidden curriculum upon established medical school identity transitions, and further study regarding the anticipatory socialization of medical school applications with the applicants themselves.

## Appendix

**Table 2:** Semi-structured focus group interview topic guide

Focus group interview topic guide	
Spine	Points to stimulate discussion
Knowledge of the hidden curriculum	Definition, difference to formal curriculum, sources of the hidden curriculum. Clinical environment sources. Educational sources.
Impact of the hidden curriculum	How different sources impact upon students plus examples. Nature of impact.
Teaching, learning and assessment within the hidden curriculum	Student opinion on the learning that occurs within the hidden curriculum, whether it is taught in any ways or should be and how it is assessed.
Other general comments	Guided by student comments.

**Table 3:** Faculty individual semi-structured interview guide

Faculty semi-structured interview guide	
Spine	Points to stimulate discussion
Understanding of the hidden curriculum	Definition, difference to formal curriculum, sources of the hidden curriculum, is it important? Are students aware of the hidden curriculum?
Examples of the hidden curriculum in action	Real-world examples of when tutors feel they have encountered or used the hidden curriculum. Impact on students.
Teaching, learning and assessment within the hidden curriculum	Tutor opinion on the learning that occurs within the hidden curriculum, whether it is taught (or they teach it) in any ways and how it is assessed. Is there anything that is difficult to teach to students?
